# Dopamine multivalent-modified polyaspartic acid for MRI-guided near-infrared photothermal therapy

**DOI:** 10.1093/rb/rbad022

**Published:** 2023-03-14

**Authors:** Liang Du, Wei Chen, Jie Zhong, Shuang Yan, Chenwu Yang, Yu Pu, Jiang Zhu, Tianwu Chen, Xiaoming Zhang, Changqiang Wu

**Affiliations:** Medical Imaging Key Laboratory of Sichuan Province and School of Medical Imaging, Affiliated Hospital of North Sichuan Medical College, Nanchong 637000, PR China; Medical Imaging Key Laboratory of Sichuan Province and School of Medical Imaging, Affiliated Hospital of North Sichuan Medical College, Nanchong 637000, PR China; Medical Imaging Key Laboratory of Sichuan Province and School of Medical Imaging, Affiliated Hospital of North Sichuan Medical College, Nanchong 637000, PR China; Medical Imaging Key Laboratory of Sichuan Province and School of Medical Imaging, Affiliated Hospital of North Sichuan Medical College, Nanchong 637000, PR China; Medical Imaging Key Laboratory of Sichuan Province and School of Medical Imaging, Affiliated Hospital of North Sichuan Medical College, Nanchong 637000, PR China; Medical Imaging Key Laboratory of Sichuan Province and School of Medical Imaging, Affiliated Hospital of North Sichuan Medical College, Nanchong 637000, PR China; Medical Imaging Key Laboratory of Sichuan Province and School of Medical Imaging, Affiliated Hospital of North Sichuan Medical College, Nanchong 637000, PR China; Medical Imaging Key Laboratory of Sichuan Province and School of Medical Imaging, Affiliated Hospital of North Sichuan Medical College, Nanchong 637000, PR China; Medical Imaging Key Laboratory of Sichuan Province and School of Medical Imaging, Affiliated Hospital of North Sichuan Medical College, Nanchong 637000, PR China; Medical Imaging Key Laboratory of Sichuan Province and School of Medical Imaging, Affiliated Hospital of North Sichuan Medical College, Nanchong 637000, PR China

**Keywords:** dopamine, superparamagnetic iron oxide, nanocomposites, magnetic resonance imaging, photothermal therapy

## Abstract

Nanophotothermal agents that provide efficient and precise treatment at tumor sites are attracting increasing attention in biomedicine. In particular, the method combination of nanophotothermal agents and magnetic resonance imaging (MRI) shows great promise for biomedical therapeutic applications. Herein, a simple nanophotothermal agent with dopamine multivalent-modified polyaspartic acid chelated superparamagnetic iron oxide (SPIO) and ferric ion (SPIO@PAsp-DAFe/PEG) was developed for MRI-guided near-infrared photothermal therapy (PTT). SPIO@PAsp-DAFe/PEG was random SPIO nanocluster with good water solubility, had a diameter of 57.8 ± 7.8 nm in dynamic light scattering, negatively charged surface (zeta potential = −11 mV), exhibited good stability and outstanding photothermal conversion efficiency (35.4%) and produced superior magnetic resonance enhanced imaging. In the experiment with tumor-bearing mice, the MRI not only monitored the accumulation of SPIO@PAsp-DAFe/PEG nanocomposites enhanced by near-infrared irradiation after intravenous administration but also determined the appropriate time window for PTT. With the use of MRI-guided near-infrared therapy, the SPIO@PAsp-DAFe/PEG nanocomposites provided excellent therapeutic effects, confirming their great potential as effective MRI/PTT therapeutic agents.

## Graphical Abstract

**Figure:**
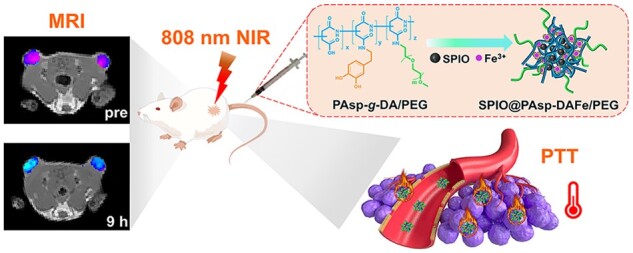

## Introduction

Cancer therapy has always been problematic for human beings [1, 2]. In recent years, building on the foundation of traditional surgery, radiotherapy and chemotherapy, new adjuvant therapies have been proposed and studied by scientists to achieve better therapeutic effects [3, 4]. Photothermal therapy (PTT) mediated by near-infrared (NIR) light is a new and promising treatment method, which utilizes photothermal conversion agents to absorb NIR light and convert the light energy into heat energy to ablate tumors while being minimally invasive, time-specific and highly effective [5, 6]. A significant number of materials have been used in the study of photothermal conversion agents, including precious-metals (Au, Ag) [7], carbon materials (graphene oxide, carbon nanotubes) [8, 9], metallic sulfides and oxides (CuS, Fe_3_O_4_) [10, 11] and organic dye substances (indocyanine green, Prussian blue) [12, 13]. However, the application of these photothermal materials remains challenging. Li *et al.* [14] successfully synthesized gold and silver hollow nanotriangles capable of achieving PTT in deep tumors, demonstrating a high photothermal conversion efficiency. It is undeniable, that precious-metal materials are high in cost and difficult to metabolize. Jayasree *et al*. [9] developed a hybrid nanosystem based on carbon dots and single-walled carbon nanotubes which not only allows fluorescence imaging but also selectively destroys cancer cells. However, the presence of excessive carbon material in the body might lead to serious lung disease. Polydopamine (PDA) has attracted considerable attention in recent years because of its excellent photothermal conversion efficiency, good biocompatibility and outstanding photothermal stability [15, 16]. Ji *et al*. [17] fabricated a mesoporous PDA-based therapeutic agent which can achieve good cancer chemo-photothermal effects under *T*_1_–*T*_2_ dual-mode MRI guidance. However, the polymerization mechanism of PDA is complex, and it is difficult to achieve controllable preparation and transformation [18]. Dopamine (DA), which is the structural monomer of PDA, is readily grafted onto polyaspartic acid in a controlled manner and then stably chelates with Fe^3+^, exhibiting an excellent photothermal effect as well [19].

Effective aggregation of photothermal agents in the tumor area is the key to successful PTT. Through medical imaging, the distribution of photothermal agents in various tissues and tumor areas of organisms can be observed in real time and non-invasively. This allows for dynamic monitoring of the tumor transmission efficiency of photothermal agents and prediction of the effect of the photothermal treatment and facilitates the selection of the best time window for laser irradiation to obtain the best photothermal treatment effect [20]. Therefore, the combination of medical imaging and photothermal agents is significant in clinical treatment. Magnetic resonance imaging (MRI) technology offers the advantages of causing no ionizing radiation, enabling better tissue contrast and allowing multi-parameter and multi-functional imaging, and has become an indispensable diagnostic imaging technology in clinical practice [21, 22]. The combination of photothermal and MRI contrast agents enables non-invasive dynamic real-time monitoring of the *in vivo* distribution of photothermal agents [23].

**Scheme 1.**
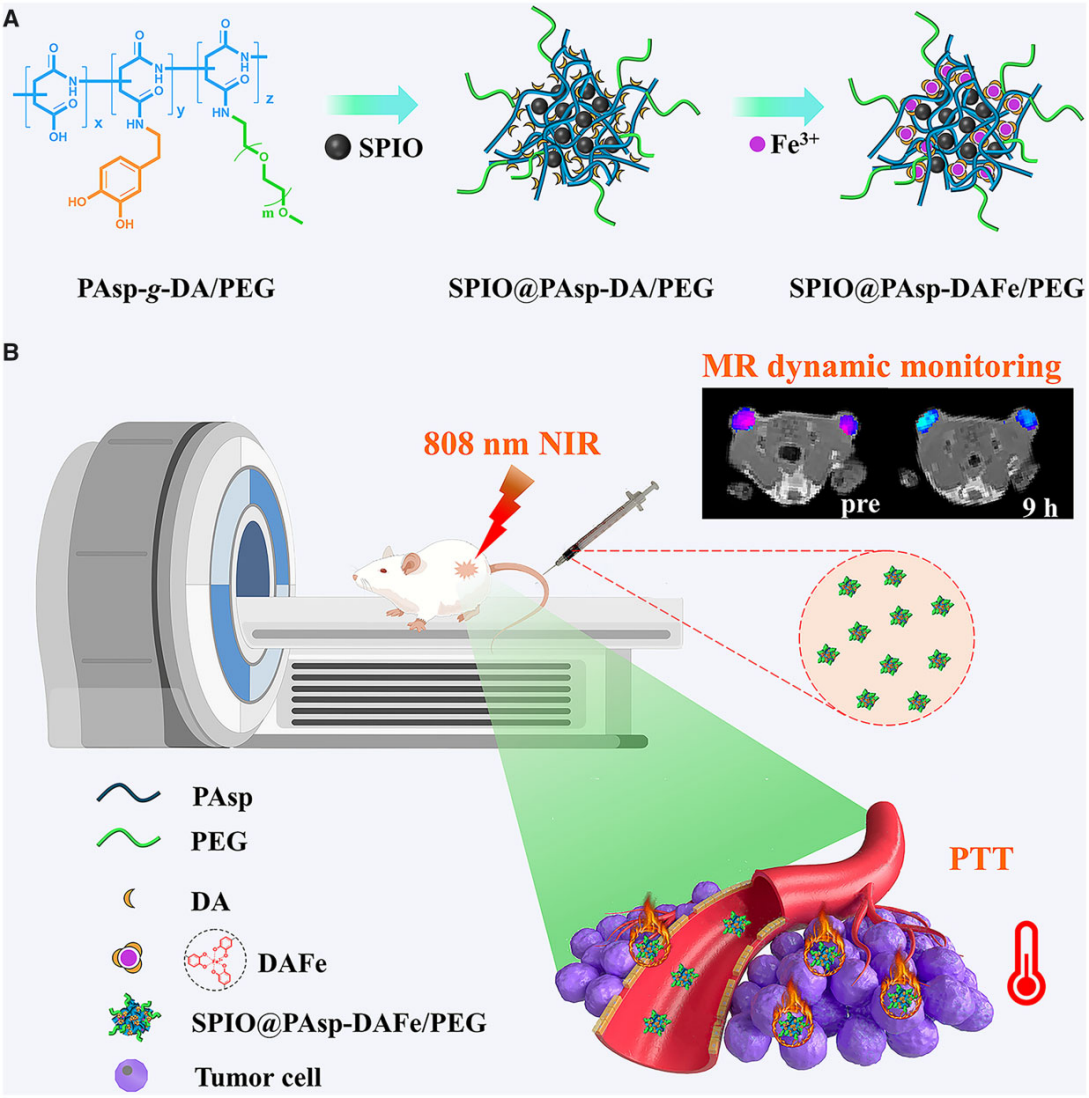
Schematic illustration of the preparation and application of SPIO@PAsp-DAFe/PEG.

Until now, most of the materials used for MRI-guided PTT required multi-step preparations, or contained large or complex structures, which limited their widespread application in cancer diagnosis and treatment [24–26]. Hence, it is significant to exploit new MRI-guided PTT agents that offer a controllable preparation using small or simple structures. In this work, a simple nanocomposite with DA multivalent-modified polyaspartic acid chelated superparamagnetic iron oxide (SPIO) and ferric ion (SPIO@PAsp-DAFe/PEG) was constructed for MRI-guided PTT. As depicted in Scheme 1, DA and polyethylene glycol grafted polyaspartic acid was used to coat the SPIO nanoparticles, and further chelate iron (III) to form bifunctional SPIO@PAsp-DAFe/PEG nanocomposites. The SPIO@PAsp-DAFe/PEG nanocomposites had the following advantages in design: (i) small-scale aggregation of SPIO nanocrystals could effectively increase *T*_2_ relaxivity and improve the MRI sensitivity. (ii) DA, a catecholamine neurotransmitter that includes catechol groups, was grafted onto PAsp in a controlled manner and displayed good photothermal conversion and photostability after chelating with ferric ions. (iii) PEG was endowed with ‘cloaking’ properties for nanoparticles, avoiding non-specific reticuloendothelial capture of nanoparticles, and ensuring the nanoparticles circulated for 24 h and accumulated efficiently within the tumor. As expected, the SPIO@PAsp-DAFe/PEG nanocomposites offered a simple nanostructure, easy preparation, good photothermal performance and good magnetic resonance contrast-imaging capability. The effect of tumor inhibition was superior to current therapies, proving these nanocomposites offer great potential in clinical application and biomedicine.

## Materials and methods

### Synthesis of PAsp-*g*-DA/PEG

All chemical reagents were used without any purification; 0.5 g poly succinimide and 0.5 g amine-terminated polyethylene glycol were fully dissolved in dimethyl sulfoxide; 0.25 g DA hydrochloride was dissolved in 1.0 ml dimethyl sulfoxide and 0.2 ml triethylamine, and then added to the reaction flask under the protection of argon gas at room temperature. The mixture was stirred at 80°C for 24 h; 0.2 g sodium hydroxide and 3.0 ml water were added to the mixture and the reaction was continued at 60°C for 24 h. Finally, the pH of the reaction solution was adjusted to 6.0 with 0.1 mol l^−1^ hydrochloric acid, and hemodialysis was performed for 24 h. The dialysate was collected and lyophilized to obtain DA and polyethylene glycol grafted polyaspartic acid (PAsp-*g*-DA/PEG). The product was verified by ^1^H NMR (BRUKER 4 mm DUI, Switzerland).

### Preparation of SPIO@PAsp-DAFe/PEG nanocomposites

Monodisperse SPIO nanoparticles in the aqueous phase were synthesized by ligand exchange reaction of hydrophobic SPIO with sodium citrate as reported previously [27]. Seven milligrams of PAsp-*g*-DA/PEG were dissolved with 14 ml ultrapure water and mixed with different content of SPIO aqueous (0.95, 0.48, 0.32 ml) (44.0 mM Fe concentration). Then the mixture was stirred at room temperature under the protection of nitrogen for 2 h. The pH of the mixture was adjusted to 8 with NaOH (0.5 M). At the end of the reaction, FeCl_3_ aqueous solution (0.074 ml, 85.5 mM) was dropped into the mixture and stirred magnetically at 1 h under the nitrogen atmosphere. Finally, the resultant solution was adjusted to neutral pH with NaOH (0.5 M) and purified through dialysis bag in ultrapure water for 6 h to obtain SPIO@PAsp-DAFe/PEG nanocomposites dispersion liquid.

### Characterization

The size and zeta potential of nanoparticles were determined with dynamic light scattering (DLS, Malvern Zetasizer Nano ZS 90, UK). The nanoparticle morphology was characterized by transmission electron microscopy (TEM, FEI Tecnai G2 F30, USA) and scanning electron microscope (SEM, ZEISS GeminiSEM 300, Germany). Fourier transform infrared (FT-IR, Thermo Scientific Nicolet 6700, USA) spectra were recorded using the KBr-pressed plates. Ultraviolet–visible–NIR (UV–vis–NIR, UV-1900i, Japan) absorption spectra were acquired in 240–900 nm wavelength. Thermo gravimetric analysis (TGA, Netzsch STA 2500, Germany) was carried out under a nitrogen environment with a heating rate of 10°C min^−1^. The Fe content in the samples was tested by inductively coupled plasma-mass spectrometry (ICP-MS, Perkin-Elmer Nexion 350×, USA).

### *In vitro* photothermal efficacy

The photothermal properties of SPIO@PAsp-DAFe/PEG nanocomposites were measured by irradiating the aqueous dispersion of nanocomposites with an 808 nm NIR laser (VCLHLGD0024991, China). The aqueous solution of SPIO, PAsp-*g*-DA/PEG, SPIO@PAsp-DA/PEG and SPIO@PAsp-DAFe/PEG was irradiated by NIR laser at a power density of 2.0 W cm^−2^ for 10 min. The SPIO@PAsp-DAFe/PEG nanocomposites aqueous solution with different graft polymer concentrations (0, 0.05, 0.1, 0.2, 0.5 mg ml^−1^) were irradiated with NIR laser (2.0 W cm^−2^) for 10 min. The PTT capabilities were detected by measuring different laser power densities (1.0, 1.5, 2.0, 3.0 W cm^−2^) of 0.2 mg ml^−1^ SPIO@PAsp-DAFe/PEG for 10 min. To detect the photothermal stability of SPIO@PAsp-DAFe/PEG nanocomposites, which were irradiated with 808 nm laser (2.0 W cm^−2^) for 10 min and then cooled naturally for 15 min cycle four times. Temperature changes of the aqueous solution of nanocomposites were recorded every 1 min.

To test the photothermal conversion efficiency (*η*) of SPIO@PAsp-DAFe/PEG nanocomposites, 0.2 mg ml^−1^ of sample solution was irradiated with an 808 nm laser (2.0 W cm^−2^) for 10 min and then the laser was turned off to allow the solution to cool naturally to the ambient temperature. The photothermal conversion efficiency was calculated by the following equation of Roper *et al*. [28]. where *h* was the heat transfer coefficient, *A* was the surface area of the material container, *T*_max_ was the maximum system temperature, *T*_amb_ was the ambient temperature, *Q*_0_ represented the heat from the light absorbed by water, *I* was the laser power and *A_λ_* was the absorbance of SPIO@PAsp-DAFe/PEG nanocomposites at 808 nm.


$$\eta_{T}=\frac{\text{hA}\left(T_{\text{max}}-T_{\text{amb}}\right)-Q_{0}}{I(1-10^{{-A}_{\lambda }})},$$

### *In vitro* MRI experiment

The relaxation times of the SPIO@PAsp-DAFe/PEG nanocomposites solution dispersed in HEPES buffer (pH = 7.4) and SPIO nanomaterials dispersed in water with different Fe concentrations (0.1, 0.2, 0.3, 0.4, 0.5 mM) were obtained at 25°C using 0.5 T (PQ001-20-015V, China), 1.41 T (minispec mq60, USA) and 3.0 T MRI systems (Discovery MR750, USA), respectively. The reciprocal of relaxation time corresponded to the concentration of Fe, and the relaxivity was determined by linear fitting. Meanwhile, these nanocomposite solutions and ultrapure water were subjected to *T*_2_-weighted MR imaging at 3.0 T. The parameters of the fast spin echo (FSE) sequence were listed as follows: TR = 3000 ms, TE = 100 ms, FOV = 180 × 180 mm^2^, matrix = 512 × 512, slice thickness = 4.0 mm and NEX = 3.0.

### *In vitro* cytotoxicity assay

The cytotoxicity of SPIO@PAsp-DAFe/PEG nanocomposites was evaluated by the Hoechst33342 assay. Briefly, EC109 cells, 4T1 cells and L929 cells were seeded onto a 96-well plate at the density of 5 × 10^3^, 5 × 10^3^ and 8 × 10^3^ cells per well, respectively. After 24 h, the medium was removed and fresh medium with different Fe concentrations (0, 5, 10, 15 and 20 μg ml^−1^) of SPIO@PAsp-DAFe/PEG nanocomposites was added to the plate. The medium was removed and Hoechst33342 was added to plates after cells were incubated at 37°C for 24 h. After 20 min of incubation at 37°C, per well was cleaned two times with PBS, the fluorescence intensity of the sample at 455 nm (excitation wavelength 352 nm) was measured with a multifunctional microplate reader (Varioskan LUX, Singapore) to calculate cell viability. The formula calculated for the relative cell viability was provided below [29]: where *A*_exp_ was the mean absorbance value of the experimental group, *A*_con_ was the mean absorbance value of the control group and *A*_blank_ was the mean absorbance value of blank group.


$$\text{Cell}\;\text{viability}\;(\%)=\frac{A_{\;\text{exp}\;}-A_{\text{blank}}}{A_{\text{con}}-A_{\text{blank}}}\times 100\%,$$

### Tumor model

The murine breast cancer cell line 4T1 was injected into the dorsally hind limb of Balb/c mice (6–8 weeks, female) to establish a subcutaneous breast cancer model. The 4T1 cell concentration was controlled from 2 × 10^6^ to 4 × 10^6^ per mice. The tumor length and width sizes were measured with a digital vernier caliper every 3 days, and the volume was calculated using the following formula: volume = length × width^2^/2 mm^3^. When tumor volumes reached 80–120 mm^3^, mice were used in MRI-guided PTT subcutaneous breast cancer therapy experiments. The experiment on mice was approved by the Animal Ethics Committee of The North Sichuan Medical College.

### *In vivo* MRI study

To examine the imaging perform *in vivo*, the SPIO@PAsp-DAFe/PEG nanocomposites (5 mg kg^−1^) were injected into the tail vein of Balb/c mice, and *T*_2_-weighted MRI examination was performed before and after injection at 3.0 T. At predetermined time points, MRI was performed *in vivo* after the injection. The tumor axial view *T*_2_ FSE and *T*_2_ map sequence parameters were shown as follows: TR = 3000 ms, TE = 104 ms, slice thickness = 1.0 mm, FOV = 60 × 42 mm^2^, matrix = 384 × 320, NEX = 2.0; TR = 3000 ms, TE = 8–68 ms, slice thickness = 1.0 mm, FOV = 80 × 80 mm^2^, matrix = 256 × 256 and NEX = 1.0.

### *In vivo* PTT therapy

After tumor volume reached 80–120 mm^3^, 4T1 tumor-bearing Balb/c mice were randomly assigned to four groups (*n* = 3): (i) saline alone; (ii) saline + NIR; (iii) SPIO@PAsp-DAFe/PEG alone; (iv) SPIO@PAsp-DAFe/PEG + NIR. One hundred microliters of saline were injected into the mice of Groups 1 and 2, while the SPIO@PAsp-DAFe/PEG was injected into Groups 3 and 4 via the tail vein with a dosage of 5 mg kg^−1^ (Fe concentration). Groups 2 and 4 received 808 nm laser treatment (1.0 W cm^−2^, 5 min) two times within half an hour, and NIR photothermal images of these two groups of mice were recorded by using a thermal imaging camera (Fotric226s, China). Following, tumor volume and body weight were monitored every 3 days for 15 days. On the 15th day, the mice were dissected and major organs were used for histological analysis.

### Histological analysis

Balb/c mice were sacrificed 15 days after treatment, and the tumor tissues and major organs (heart, liver, spleen and kidney) were collected from treatment groups. Then, they were stained with hematoxylin–eosin (H&E) and observed under the optical microscope for histopathological analysis. Prussian blue staining was performed on major organs (liver, spleen, kidney and tumor) before and after the injection of nanocomposites.

### Statistical analysis

The data are presented as mean ± standard deviation (SD), and the significance of the data in this work was analyzed according to Student’s *t*-test (*P <* *0.05).

## Results and discussion

### Characterization of SPIO@PAsp-DAFe/PEG nanocomposites

**Figure 1.**
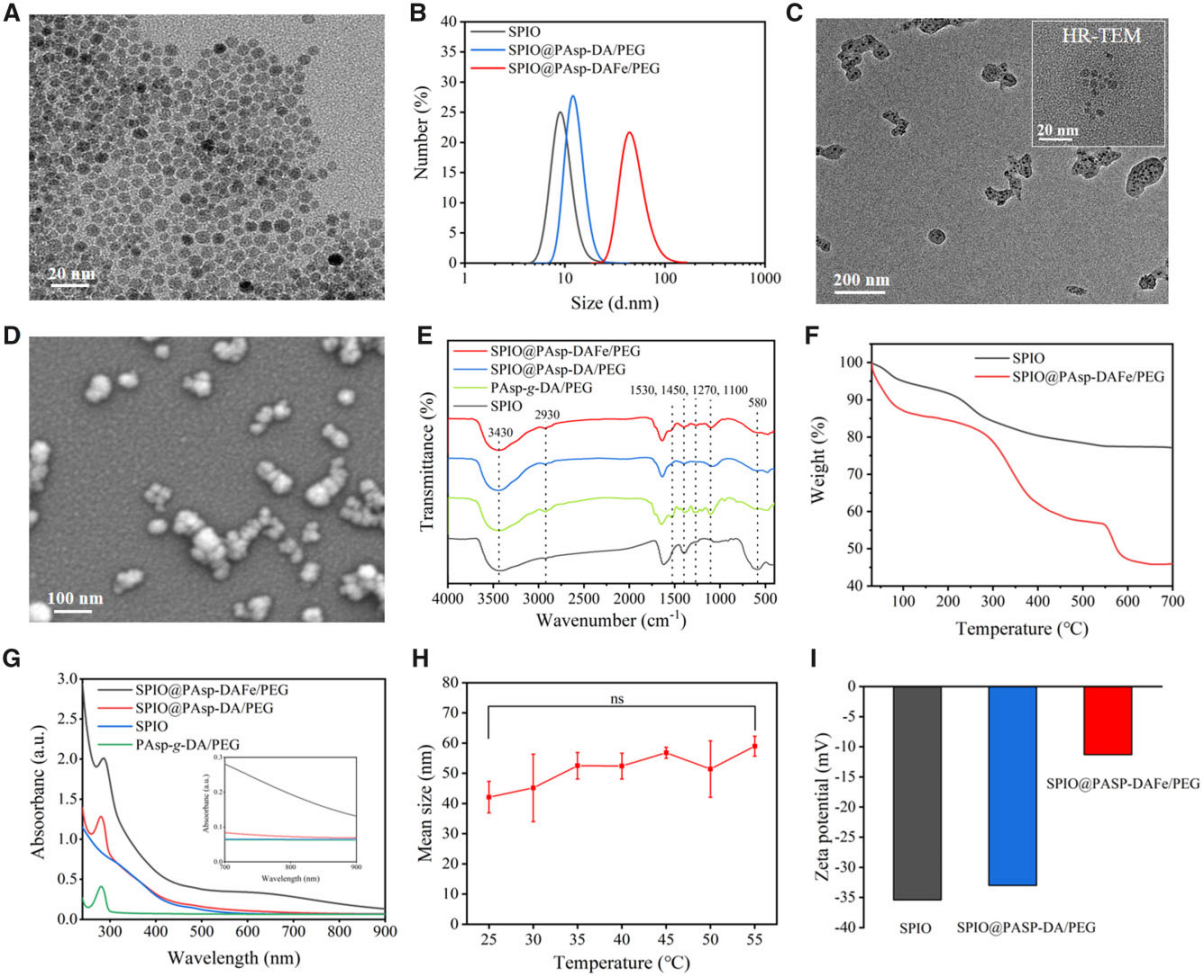
Characterizations of SPIO@PAsp-DAFe/PEG nanocomposites. (**A**) TEM image of citric acid-coated SPIO nanocrystals. (**B**) Hydrodynamic sizes of SPIO, SPIO@PAsp-DA/PEG, and SPIO@PAsp-DAFe/PEG nanocomposites aqueous solution with DLS. (**C**) TEM image of SPIO@PAsp-DAFe/PEG nanocomposites. (**D**) SEM image of SPIO@PAsp-DAFe/PEG nanocomposites. (**E**) FT-IR spectra of SPIO, PAsp-*g*-DA/PEG, SPIO@PAsp-DA/PEG and SPIO@PAsp-DAFe/PEG nanocomposites. (**F**) TGA analysis of SPIO and SPIO@PAsp-DAFe/PEG nanocomposites. (**G**) UV–vis–NIR spectra of SPIO, PAsp-*g*-DA/PEG, SPIO@PAsp-DA/PEG and SPIO@PAsp-DAFe/PEG nanocomposites. (**H**) Hydrodynamic sizes of SPIO@PAsp-DAFe/PEG nanocomposites aqueous solution at different temperatures (ns is non-significant). (**I**) Zeta-potential of the aqueous solution of SPIO, SPIO@PAsp-DA/PEG and SPIO@PAsp-DAFe/PEG nanocomposites.

DA and polyethylene glycol grafted polyaspartic acid (PAsp-*g*-DA/PEG) was synthesized by the ammonolysis of poly succinimide (PSI) and was verified by ^1^H NMR spectroscopy (Supplementary Fig. S1). The characteristic peaks of DA (3, 4, 5) and PEG (6, 7) appeared in the ^1^H NMR spectra of PAsp-*g*-DA/PEG, indicating that DA and PEG were successfully grafted onto the polyaspartic acid side chain. SPIO nanocrystals were synthesized in a high-temperature thermal decomposition process and transferred to an aqueous phase by a ligand exchange reaction with citric acid. Homogeneous dispersibility was confirmed by TEM image analysis (Fig. 1A). DLS measurements indicated that the average particle size of the SPIO nanocrystals was 6 nm and the distribution was narrow, which was beneficial to post-modification (Fig. 1B). The SPIO@PAsp-DAFe/PEG nanocomposites were prepared by coating SPIOs and chelating trivalent iron ions with PAsp-*g*-DA/PEG, as illustrated in Scheme 1. In the experiment, three ratios (by mass) of polymer to SPIO (3:1, 2:1, 1:1) were used for the preparation of SPIO@PAsp-DAFe/PEG nanocomposites. The surface potential of the SPIO@PAsp-DAFe/PEG nanocomposites was negative, and the mean diameters gradually increased with an increase in the proportion of SPIO (Supplementary Figs S2 and S3). The UV–vis–NIR absorption spectrum revealed that when the mass ratio of polymer to SPIO was 2:1, the SPIO@PAsp-DAFe/PEG nanocomposites exhibited the strongest NIR absorption at 808 nm in the same conditions (Supplementary Fig. S4). Hence, the PAsp-*g*-DA/PEG to SPIO mass ratio of 2:1 was selected for subsequent experiments because of its suitable size (57.8 ± 7.8 nm) and strong absorption in the NIR region.

The diameter of the SPIO@PAsp-DAFe/PEG nanocomposites was significantly greater than that of the SPIO nanocrystals, indicating that the SPIO surface was surrounded by polymers (Fig. 1B). The TEM and SEM images of the SPIO@PAsp-DAFe/PEG nanocomposites (Fig. 1C and D) also showed that the SPIO was successfully encapsulated inside the polymers and formed random SPIO nanoclusters. The energy-dispersive X-ray spectroscopy analysis revealed the elemental chemistry of the SPIO@PAsp-DAFe/PEG nanocomposites composition, specifically the presence of C, N, O and Fe (Supplementary Fig. S5). The FT-IR spectra of SPIO, PAsp-*g*-DA/PEG, SPIO@PAsp-DA/PEG and the SPIO@PAsp-DAFe/PEG nanocomposites were displayed in Fig. 1E. The absorption peak at 580 cm^−1^ was related to the Fe–O vibration. The vibration peak of the Fe–O bond of SPIO@PAsp-DAFe/PEG nanocomposites was weakened, indicating that SPIO was successfully encapsulated [30]. The absorption bands at ∼1270, 1530 and 1450 cm^−1^ were associated with the catechol structure [31, 32]. The bands at 1100, 2930 and 3440 cm^−1^ corresponded to the stretching vibration of the C–O bond, the alkyl (CH_2_) chain, and the O–H bond in PEG, respectively [33]. These results further demonstrated the successful construction of the SPIO@PAsp-DAFe/PEG nanocomposites. The TGA of the nanocomposites was shown in Fig. 1F. The weight loss of the SPIO nanoparticles was ∼22.9% at 700°C. After modifying the polymer on the SPIO nanoparticles, the weight loss of nanocomposites was increased to 54.1%, indicating SPIO nanoparticles could be coated with the polymer. The SPIO@PAsp-DAFe/PEG nanocomposites exhibited extensive absorption from visible light to the NIR region compared with SPIO, PAsp-*g*-DA/PEG, and SPIO@PAsp-DA/PEG (Fig. 1G). The SPIO@PAsp-DAFe/PEG nanocomposites showed significantly higher NIR absorption, which could be attributed to the chelate of DA with Fe^3+^. To evaluate the stability of the SPIO@PAsp-DAFe/PEG nanocomposites with increasing temperature, we investigated the DLS of the nanocomposites in the temperature range of 20–55°C. As shown in Fig. 1H, the particle size of the hydrated nanocomposites showed minimal change with an increase in temperature, indicating that the SPIO@PAsp-DAFe/PEG nanocomposites were stable. In addition, zeta-potential measurements showed that the potential of the SPIO@PAsp-DAFe/PEG nanocomposites (−11 mV) was lower than that of SPIO (−35 mV) and SPIO@PAsp-DA/PEG (−33 mV) (Fig. 1I). The negative charge was likely derived from the carboxyl group on the PAsp block and the catechol group of DA on the surface of the nanocomposites. The addition of iron ions resulted in cross-linking within the nanocomposites, which lead to a decrease in the surface potential.

### MRI performance of SPIO@PAsp-DAFe/PEG nanocomposites *in vitro*

**Figure 2.**
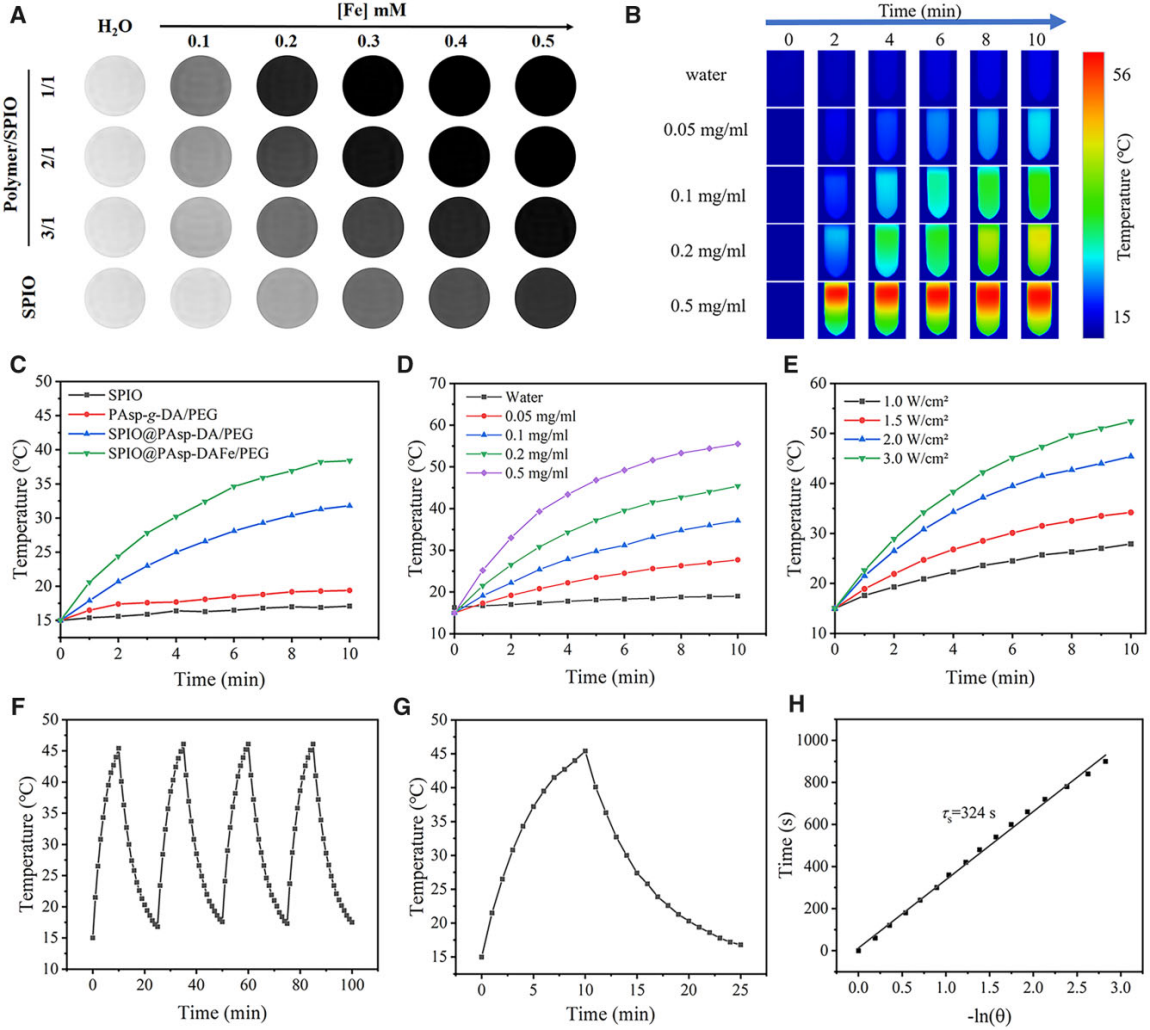
MRI and photothermal properties of SPIO@PAsp-DAFe/PEG nanocomposites. (**A**) *In vitro T*_2_-weighted MRI images of SPIO and different proportions of SPIO@PAsp-DAFe/PEG nanocomposites solution with different concentrations at 3.0 T (FSE, TR = 3000 ms, TE = 100 ms, FOV = 180 × 180 mm^2^, matrix = 512 × 512, slice thickness = 4.0 mm, NEX = 3.0). (**B**) The thermal infrared images of SPIO@PAsp-DAFe/PEG nanocomposites aqueous solution with different concentrations under 808 nm laser (2.0 W cm^−2^). (**C**) Photothermal heating curves of SPIO, PAsp-*g*-DA/PEG, SPIO@PAsp-DA/PEG and SPIO@PAsp-DAFe/PEG nanocomposites aqueous solution irradiated for 10 min by 808 nm laser. (**D**) Photothermal heating curves of SPIO@PAsp-DAFe/PEG nanocomposites at different concentrations irradiated for 10 min by 808 nm laser. (**E**) Laser power dose-dependent temperature elevation of SPIO@PAsp-DAFe/PEG nanocomposites aqueous solution (0.2 mg ml^−1^) under 808 nm laser for 10 min. (**F**) The temperature variations of SPIO@PAsp-DAFe/PEG nanocomposites aqueous solution using laser irradiation for four cycles. (**G**) The photothermal response of the SPIO@PAsp-DAFe/PEG nanocomposites aqueous solution at the whole heating and cooling process. (**H**) Plots of cooling time (15 min) and the negative natural logarithm of the temperature driving force obtained from the cooling stage. The time constant of sample system *τ*_s_ was 324 s.

To evaluate the ability of the MRI contrast agent to enhance imaging, the longitudinal (*T*_1_) and transverse (*T*_2_) relaxation times of the SPIO@PAsp-DAFe/PEG solution (different proportions of polymers/SPIO) and the SPIO solution at 0.5, 1.41 and 3.0 T were studied by inversion recovery sequence and the spin-echo pulse sequence. As listed in Supplementary Table S1, the size of nanocomposites and transverse relaxivity (*r*_2_) values increased with a decrease in the polymer/SPIO ratio at the same magnetic-field intensity. However, the *r*_2_ values and *r*_2_/*r*_1_ ratio of the SPIO@PAsp-DAFe/PEG nanocomposites solution increased with an increase in magnetic-field intensity. This phenomenon indicated that *r*_2_ was more effective at higher fields, reflecting the ability of the SPIO@PAsp-DAFe/PEG nanocomposites solution to generate local magnetic inhomogeneities. For a given material, the *r*_2_/*r_1_* ratio is an important reference for evaluating the properties of *T*_1_- or *T*_2_-weighted MRI contrast agents. In general, the *T*_2_ contrast agent had a relatively higher *r*_2_/*r_1_* ratio (>8) than that of the *T*_1_ contrast agent (<5) [34]. The *r*_2_/*r_1_* ratio of the SPIO@PAsp-DAFe/PEG nanocomposites solution (polymer/SPIO = 2:1) was 36.8, suggesting that the nanocomposites could be used as a *T*_2_-weighted MRI contrast agent. In addition, the *r*_2_ value of the SPIO@PAsp-DAFe/PEG nanocomposites solution (92.09 mM^−1^ s^−1^) was higher than that of the pristine SPIO nanocrystals solution, indicating that the SPIO@PAsp-DAFe/PEG nanocomposites acted as a better *T*_2_-weighted MRI contrast candidate. As shown in Fig. 2A, the *T*_2_ signal intensity of the nanocomposite solution decreased significantly with an increase in iron concentration, as the nanoparticles distort the magnetic field more efficiently, generating darker images [35]. The phenomenon suggested that the SPIO@PAsp-DAFe/PEG nanocomposites could produce clear *T*_2_-weighted MR contrast at 3.0 T, which was expected to be a promising candidate for a *T*_2_-weighted MRI contrast agent.

### Photothermal properties of SPIO@PAsp-DAFe/PEG nanocomposites *in vitro*

Due to the insufficient ability of NIR light to penetrate tissue, photothermal conversion agents were used to improving PTT efficiency. The NIR photothermal conversion of SPIO@PAsp-DAFe/PEG nanocomposites dispersed in water was further investigated. Visualizing the temperature changes in SPIO@PAsp-DAFe/PEG nanocomposites was enabled by infrared thermal imaging (Fig. 2B). PAsp-*g*-DA/PEG, SPIO and SPIO@PAsp-DA/PEG were imaged and compared under the same conditions (808 nm, 2.0 W cm^−2^, 10 min). As shown in Fig. 2C, the temperature of an aqueous solution of SPIO@PAsp-DAFe/PEG increased from 15°C to 38.4°C, compared with SPIO, which increased to 17.1°C, PAsp-*g*-DA/PEG which increased to 19.4°C and SPIO@PAsp-DA/PEG which increased to 31.8°C. Subsequently, 1 ml of varying concentrations of the SPIO@PAsp-DAFe/PEG nanocomposite aqueous solution was irradiated by an 808 nm laser. After continuous irradiation for 10 min, the temperature of the 0.2 mg ml^−1^ SPIO@PAsp-DAFe/PEG aqueous solution increased by 30.4°C, whereas the temperature of water increased by only 2.7°C. Photothermal conversion, which is vital for hyperthermia to kill tumor cells, showed a strong dependence on concentration (Fig. 2D). The photothermal performance of the SPIO@PAsp-DAFe/PEG nanocomposites was also highly dependent on the NIR light energy density, and the final temperature increased as the laser power increased at the same heating time (Fig. 2E). Furthermore, under an 808 nm laser (2.0 W cm^−2^), the photothermal performance of the SPIO@PAsp-DAFe/PEG nanocomposites did not change significantly for four cycles, indicating outstanding photothermal stability (Fig. 2F). According to Fig. 2G and H, the photothermal conversion efficiency of SPIO@PAsp-DAFe/PEG nanocomposites was 35.4%, which was much higher than that of gold nanorods (27.1%), graphene oxide NPs (13.9%) and copper sulfide NPs (27.2%) [36–38]. The results indicate that the SPIO@PAsp-DAFe/PEG nanocomposites were excellent at photothermal conversion and were optically stable, which makes them promising candidates for PTT applications.

### Serum stability and cytotoxicity of SPIO@PAsp-DAFe/PEG nanocomposites *in vitro*

**Figure 3.**
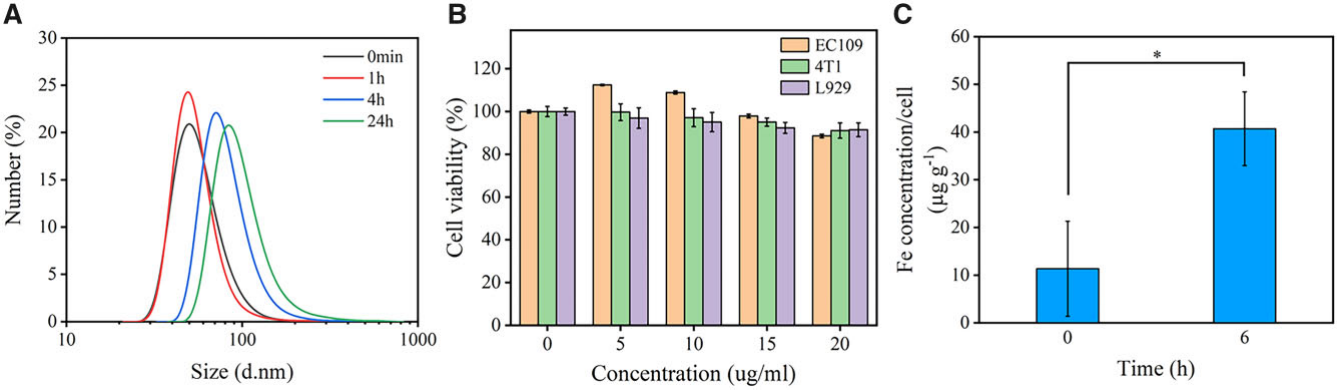
Biological characteristics of SPIO@PAsp-DAFe/PEG nanocomposites. (**A**) DLS analysis of SPIO@PAsp-DAFe/PEG nanocomposites and a 20% (v/v) FBS solution co-incubated for 24 h. (**B**) The viability of EC109 cells, 4T1 cells and L929 cells after incubating with different concentrations of SPIO@PAsp-DAFe/PEG nanocomposites for 24 h. (**C**) Cellular uptake quantitative assay of the SPIO@PAsp-DAFe/PEG nanocomposites after 6 h incubation (*P <* *0.05).

To further evaluate the potential stability *in vivo*, after incubation in a 20% (v/v) FBS solution at 37°C for 24 h, the size changes in SPIO@PAsp-DAFe/PEG nanocomposites in DLS were monitored. Figure 3A revealed that there was almost no change in the size of the SPIO@PAsp-DAFe/PEG nanocomposites within 1 h of co-incubation, in spite of nanocomposites being immediately covered by proteins. However, minor changes in size were observed in the SPIO@PAsp-DAFe/PEG nanocomposites after 4 h, and a dynamic protein-absorption process seemed to occur for SPIO@PAsp-DAFe/PEG nanocomposites, though there was no visible precipitation throughout the process [39, 40]. To evaluate the *in vitro* cytotoxicity of the SPIO@PAsp-DAFe/PEG nanocomposites, the Hoechst33342 assay was used to analyze the viability of cells (EC109 cells, 4T1 cells and L929 cells) incubated with SPIO@PAsp-DAFe/PEG nanocomposites for 24 h. The results suggested that the cells still maintained strong cellular activity (>90%) (Fig. 3B), indicating the low cytotoxicity of the SPIO@PAsp-DAFe/PEG nanocomposites. In addition, we quantitatively assessed the cytophagy of SPIO@PAsp-DAFe/PEG nanocomposites by ICP-MS. As displayed in Fig. 3C, before and after the incubation of 4T1 cells and the SPIO@PAsp-DAFe/PEG nanocomposites, the intracellular iron content (dry weight) was 11.3 and 40.7 µg g^−1^ (Fe mass per gram cells), respectively, which proved that only a small amount of iron was taken in by the cells.

### The dynamic analysis accumulation of SPIO@PAsp-DAFe/PEG nanocomposites in the tumor area by MRI

**Figure 4.**
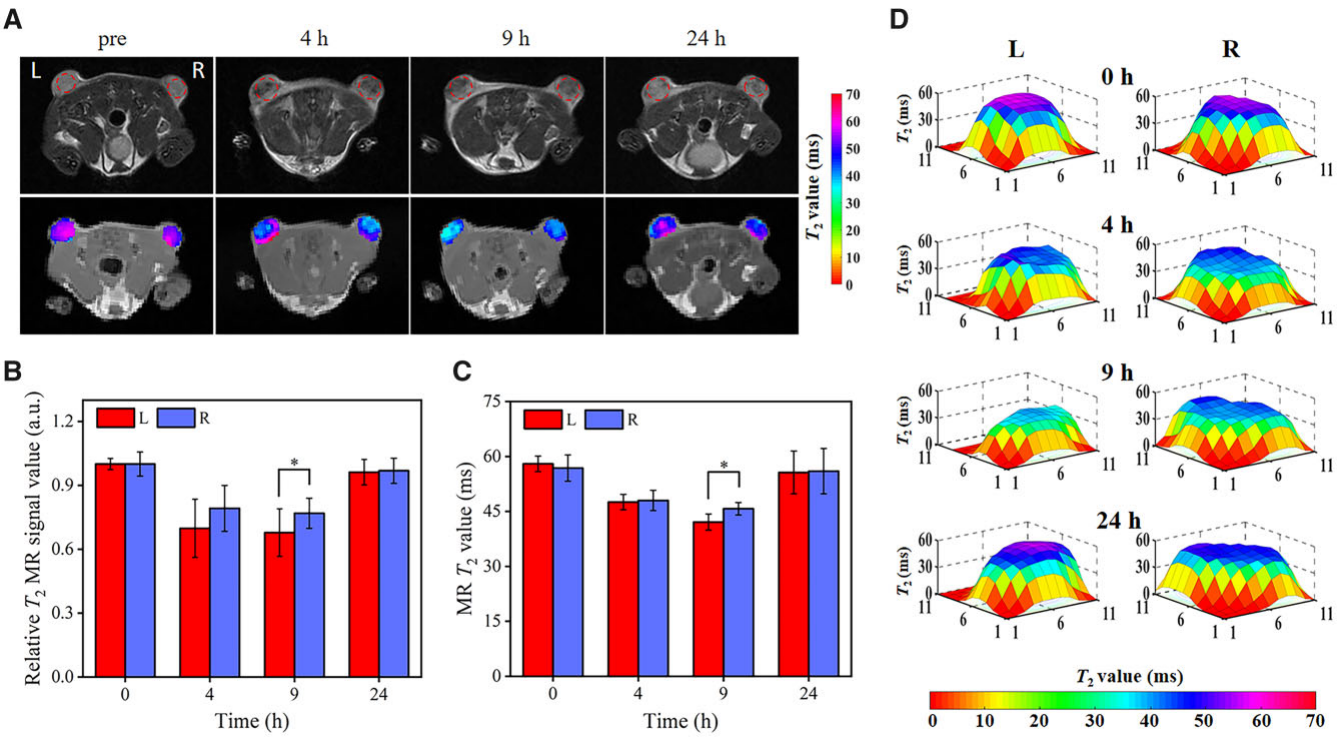
*In vivo* tumor MRI. *In vivo T*_2_-weighted images, *T*_2_ map (**A**) and corresponding relative *T*_2_ MR signal (**B**), *T*_2_ value (**C**) of 4T1 tumor-bearing Balb/c mice with (L, left) and without (R, right) laser irradiation, in the axial position, after SPIO@PAsp-DAFe/PEG nanocomposites injection (5 mg kg^−1^). (**D**) Tumor *T*_2_ value distribution after intravenous SPIO@PAsp-DAFe/PEG nanocomposites injection (*P <* *0.05).

In order to verify whether MRI can be used to noninvasively and effectively monitor the accumulation of SPIO@PAsp-DAFe/PEG nanocomposites in the tumor region, tumors were observed under a 3.0 T MRI system before and 4, 9 and 24 h after intravenous administration. The concentration of the SPIO@PAsp-DAFe/PEG nanocomposites that accumulated in the tumor area was reflected in the intensity of the MR signal. The use of thermal delivery techniques at appropriate temperatures selectively increased the perfusion and permeability of tumor vasculature, thus enabling the delivery of nanomedicines under laser radiation [41]. Thus, we designed a control experiment with and without NIR laser irradiation. First, a tumor on the left side of a mouse was heated with a NIR laser to ∼42°C for 10 min immediately after intravenous administration (Supplementary Fig. S6). The tumors would have some cancer-specific cytotoxicity at that temperature, resulting in increased tumor vascular perfusion [41]. On the contrary, the tumor was not irradiated with NIR on the right side of the mice. As shown in Fig. 4A and B, signals at the tumor site before intravenous injection were high. After intravenous administration, the tumor signals on the left and right sides of the mice gradually decreased with increasing time reaching their lowest at 9 h, indicating that the concentration of the SPIO@PAsp-DAFe/PEG nanocomposites at the tumor site was at its greatest at this moment. Encouragingly, the reduction in relative *T*_2_ MR signal value was more pronounced on the left side than on the right at 9 h, depending on time and heating. The *T*_2_-map quantitative analysis further revealed that the mean *T*_2_ value decreased by 27% for left-sided tumors, from 57.9 ± 2.1 to 42.1 ± 2.2 ms, and by 19% for right-sided tumors, from 56.8 ± 3.5 to 45.7 ± 1.7 ms. These results demonstrate that heat transfer techniques at appropriate temperatures can effectively increase the aggregation of SPIO@PAsp-DAFe/PEG nanocomposites at the tumor site (Fig. 4C and D).

### Photothermal treatment of tumors under MRI-guidance

**Figure 5.**
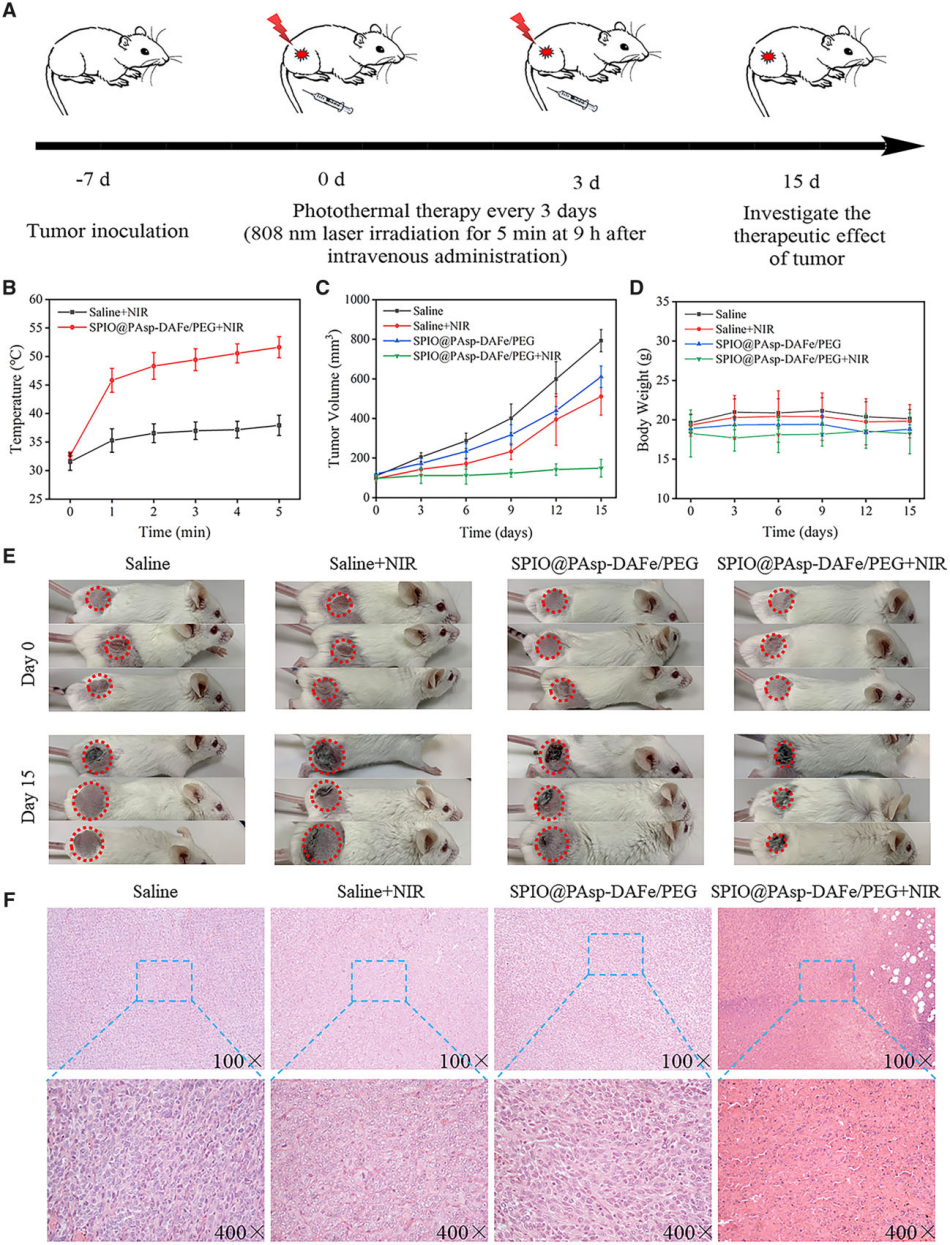
*In vivo* antitumor therapy. (**A**) Tumor treatment options in 4T1 tumor-bearing Balb/c mice. (**B**) The tumor temperature was recorded under laser irradiation after saline and SPIO@PAsp-DAFe/PEG nanocomposites injection (*n* = 3). (**C**) The tumor growth curves of mice after different treatments during the PTT period. (**D**) The body weight of the mice was recorded after different treatments during the PTT period. (**E**) Digital photos of tumor regions in 4T1 tumor-bearing Balb/c mice on Day 0 and 15 after treatment. (**F**) 4T1 tumor-bearing Balb/c mice were sacrificed on Day 15 after different treatments for H&E-stained of histological sections.

We further evaluated the antitumor ability and efficacy of SPIO@PAsp-DAFe/PEG nanocomposites in 4T1 tumor-bearing Balb/c mice, based on the ideal effect of photothermal conversion *in vitro*. Intravenous administration was performed when the tumor reached 80–120 mm^3^, at which point the tumor was immediately irradiated with an 808 nm laser to maintain a temperature of ∼42°C for 10 min. The tumor was again irradiated twice using the laser when the SPIO@PAsp-DAFe/PEG nanocomposites were circulated *in vivo* after 9 h. Increasing the irradiation time may burn the surrounding normal cells (Fig. 5A). As depicted in Supplementary Fig. S7, the whole-mice body thermal imaging taken at different times showed that the temperature at the tumor site rapidly increased with increasing irradiation time. In contrast, almost no change in temperature was detected during NIR laser irradiation without the SPIO@PAsp-DAFe/PEG nanocomposites. The temperature of the tumor regions was rapidly raised to ∼51.6°C for 5 min with the SPIO@PAsp-DAFe/PEG nanocomposites during NIR laser irradiation (Fig. 5B), which was sufficient to eliminate cancer cells. In contrast, during NIR laser irradiation of tumors injected with saline, the temperature of the tumor site only reached ∼37.9°C. This demonstrates the excellent light-to-heat transformation efficiency of the SPIO@PAsp-DAFe/PEG nanocomposites *in vivo*.

To assess the antitumor effect of PTT, tumor volume and mice body weight of the four groups were measured and recorded every 3 days. Fig. 5C shows that the tumor volume of the SPIO@PAsp-DAFe/PEG + NIR group was significantly suppressed after 15 days of treatment while saline, saline + NIR or SPIO@PAsp-DAFe/PEG alone showed large-volume tumors. Importantly, the mice in the four groups emerged with negligible fluctuations in body weight throughout the treatment (Fig. 5D), demonstrating that the SPIO@PAsp-DAFe/PEG nanocomposites had no significant side effects. Fig. 5E shows the results of four groups (*n* = 3) of mice treated with different methods, on Days 0 and 15. The tumors treated with the SPIO@PAsp-DAFe/PEG nanocomposites were smaller after light exposure, and significant burn scars appeared at the tumor sites. This suggests that SPIO@PAsp-DAFe/PEG + NIR effectively inhibited tumor growth without affecting body weight.

In order to further demonstrate the efficacy of PTT using SPIO@PAsp-DAFe/PEG nanocomposites, sections of H&E staining tumor were taken from different treatment groups, and histological analysis was performed (Fig. 5F). Significantly, the tumors treated with the SPIO@PAsp-DAFe/PEG nanocomposites + NIR showed signs of cell necrosis, while tumors in the other three groups (saline only, saline + NIR and SPIO@PAsp-DAFe/PEG nanocomposites only) showed almost complete tissue structure. These phenomena revealed that the SPIO@PAsp-DAFe/PEG nanocomposites are an effective form of PTT with the capability of *T*_2_-weighted imaging.

### Histological and toxicology assessment *in vivo*

Encouraged by the prominent anticancer effect and superior MRI-enhanced capabilities of SPIO@PAsp-DAFe/PEG nanocomposites, we performed histological and toxicological evaluations on mice. The major organs of 4T1 tumor-bearing Balb/c mice were stained with Prussian blue before and 4, 9 and 24 h after intravenous administration of the SPIO@PAsp-DAFe/PEG nanocomposites. Supplementary Fig. S8 shows that a large number of blue-stained particles were found in the liver, spleen and tumor, but not in the kidney, indicating that the nanocomposites were mainly metabolized through the liver and spleen. Blue-stained particles were widely distributed in the liver, spleen and tumor at 4 and 9 h, and decreased at 24 h, indicating that some of the nanocomposites had been metabolized. The nanocomposites could effectively accumulate in the tumor due to the abundance of blood vessels at the tumor site and the prolonged circulation of nanocomposites in the body, giving the tumor enough of the nanocomposites to reach the required temperature for PTT.

**Figure 6.**
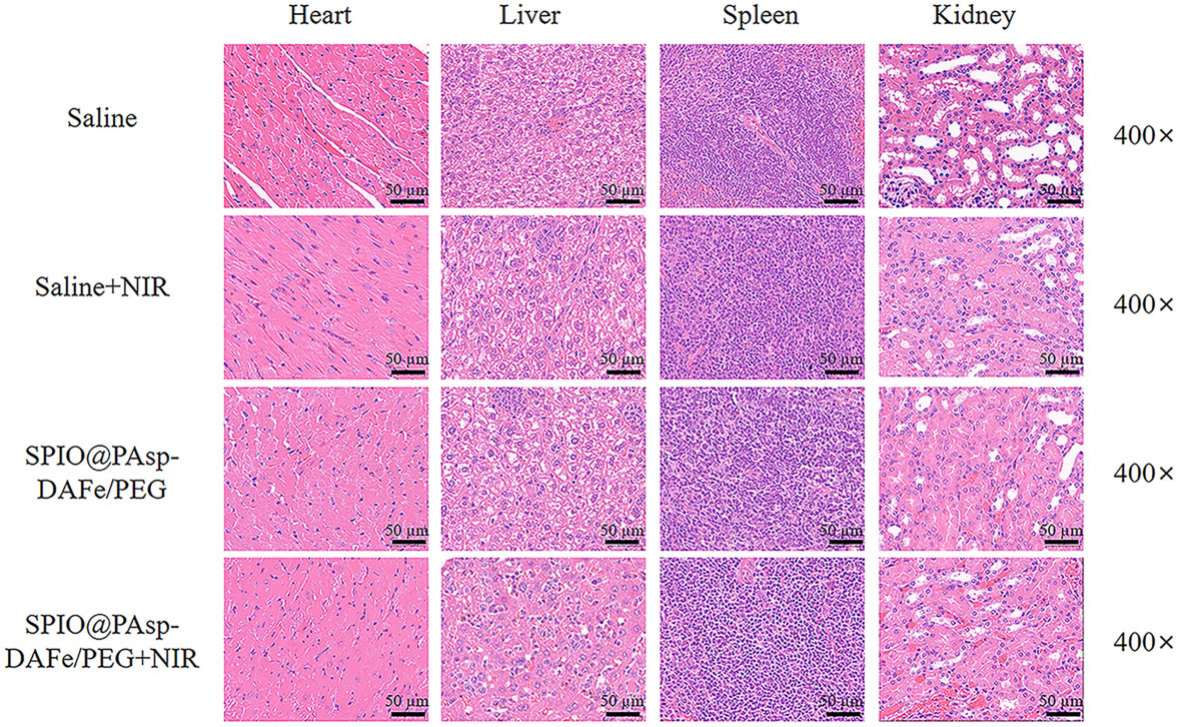
H&E staining histological slides of heart, liver, spleen and kidney from the 4T1 tumor-bearing Balb/c mice after various treatments and sacrificed on Day 15.

A total of 15 days after photothermal treatment of the tumor, all mice were euthanized to harvest their main organs for H&E staining. As shown in Fig. 6, there was no significant tissue abnormality or damage to the heart, liver, spleen or kidney of the mice in all groups, indicating negligible long-term adverse toxicity of the major organs. *In vivo*, acute toxicity was assessed in healthy Balb/c mice (*n* = 4) after intravenous administration of 20 mg kg^−1^ of the SPIO@PAsp-DAFe/PEG nanocomposites, while the control group was injected with saline. There were no significant differences in food and water consumption, excretion, daily behavior and body weight between the control group and the mice treated with the SPIO@PAsp-DAFe/PEG (Supplementary Fig. S9). Deaths or apparent clinical complications were not observed. The results indicated that the SPIO@PAsp-DAFe/PEG nanocomposites were biocompatible, suggesting they may be useful in additional biological applications.

## Conclusion

In summary, we successfully synthesized a simple nanoscale photothermal therapeutic agent with MRI enhancement based on DA-grafted polyaspartic acid and SPIO using a simple method, a concise, efficient method that resulted in low toxicity. The SPIO@PAsp-DAFe/PEG nanocomposites that were obtained with a suitable particle size (57.8 ± 7.8 nm) revealed a higher *T*_2_ relaxivity and NIR photothermal conversion efficiency, as well as good stability and repeatability. The distribution of nanocomposites in the tumor area was effectively monitored using MRI, which allowed a precise tumor treatment protocol to be developed for PTT. More importantly, the SPIO@PAsp-DAFe/PEG nanocomposites inhibited tumor growth in mice without significant signs of toxicity, thus making it an effective *T*_2_-weighted MRI/PTT therapeutic agent. Although the 808 nm wavelength NIR laser penetrated to a limited depth of tissue, MRI-guided PTT based on SPIO@PAsp-DAFe/PEG nanocomposites can be used for treatment of the superficial tumors (e.g. breast cancer) or lumen tumors (e.g. cervical cancer, vaginal cancer, nasopharyngeal cancer, esophageal cancer, rectal cancer). Overall, the work not only highlighted the translational potential of SPIO@PAsp-DAFe/PEG nanocomposites for biologic applications but also established a new therapeutic approach using MRI guidance.

## Supplementary Material

rbad022_Supplementary_Data
