# Supplementary material for: Dopamine multivalent-modified polyaspartic acid for MRI-guided near-infrared photothermal therapy
Source: Regen Biomater. 2023 Mar 14;10:rbad022. doi: 10.1093/rb/rbad022 (PMC10097457; doi:10.1093/rb/rbad022)

**Supporting Information**

**Dopamine multivalent-modified polyaspartic acid for MRI-guided near-infrared photothermal therapy**

Liang Du, Wei Chen, Jie Zhong, Shuang Yan, Chenwu Yang, Yu Pu, Jiang Zhu, Tianwu Chen, Xiaoming Zhang* and Changqiang Wu*

Medical Imaging Key Laboratory of Sichuan Province and School of Medical Imaging, Affiliated Hospital of North Sichuan Medical College, Nanchong 637000, P. R. China.

*Correspondence address. Sichuan Key Laboratory of Medical Imaging, North Sichuan Medical College, 234th Fujiang Rd., Nanchong 637000, China. E-mail: wucq1984@nsmc.edu.cn (C.W.); zhangxm@nsmc.edu.cn (X.Z.)

**S1. Experimental section**

**S1.1. Chemicals**

Iron (Ⅲ) acetylacetonate (Fe(acac)_3_, 99%), 1,2-hexadecanediol (90%), oleic acid (90%), oleylamine (70%), phenyl ether, citric acid, sodium hydroxide, hexane, and acetone were purchased from Sigma-Aldrich. Polysuccinimide (PSI, MW = 8000-10000) were purchased from Mackline. Dopamine hydrochloride (98%) were purchased from Innochem. All chemicals were used as received without further purification. Ultrapure water (18 MΩ/cm) in all experiments was obtained by passing through an ultra-pure purification system.

**S1.2. Synthesis of hydrophobic SPIO nanocrystals**

Fe(acac)_3_ (1 mM), 1, 2-hexadecanediol (5 mM), oleic acid (3 mM), oleylamine (3 mM), and phenyl ether (10 mL) were mixed and magnetically stirred under a flow of nitrogen after deoxidized in a two-necked flask. The mixture was heated to 200 °C for 30 min in diatomaceous earth. Under continuous nitrogen, the mixture was heated to reflux (256 °C) for 30 min. After removing the heat source, the mixture cooled to room temperature. Ethanol (160 mL) was added to the mixture to precipitate the black material and centrifuged (5500 rpm, 10 min) for separation. The black product was dissolved in a small amount of hexane and centrifuged (12000 rpm, 20 min) to remove any undispersed residue. Again, the black product was then precipitated with ethanol, centrifuged (5500 rpm, 10 min) to remove the solvent, and dispersed into a small amount of hexane. At this point, the hydrophobic SPIO was successfully synthesized.

**S1.3. Synthesis of hydrophilic SPIO nanocrystals**

Hydrophobic SPIO (6 ml), citric acid (3.7 mM), sodium hydroxide (11.2 mM), ultrapure water (70 mL), hexane (70 mL), and acetone (110 mL) were mixed and magnetically stirred under a condition of nitrogen and oil bath heated to 70 °C condensation reflux for 48 h after deoxidizing in a flask. Removing the heating source and the mixture cools naturally to room temperature. Ethanol (160 mL) was added to the mixture to precipitate the material and centrifuged (5000 rpm, 10 min) for separation. The product was dissolved in a small amount of ultrapure water to obtain hydrophilic SPIO.

**S1.4. Calculation of photothermal conversion efficiency of SPIO@ PAsp-DAFe/PEG nanocomposites**

The photothermal conversion efficiency (*η*) of SPIO@PAsp-DAFe-PEG was calculated using following equations：

(1)

$$\eta=\frac{hs\left（ T_{max}-T_{amb} \right）-Q_{0}}{I(1-{10}^{-A})}$$

In equal (1), in order to get *hs*, *θ* as a dimensionless driving force temperature is introduced:

(2)

$$\theta=\frac{T-T_{amb}}{T_{max}-T_{amb}}$$

*Q*_0_ is the heat loss caused by the light absorbed by the solvent and ep tubes:

(3)

$$Q_{0}=hs{(T}_{max(water)}-T_{amb})$$

*τ*s is introduced as a sample system constant:

(4)

$$\tau_{s}=\frac{mC_{water}}{hs}$$

Integrating eq (2), (3) and (4):

(5)

$$t=-\tau_{s}\ln\theta=-\frac{mC_{water}}{hs}\ln\theta$$

As shown in Figure 3E, *τ*s =324.5s

$$hs=\frac{1\times4.2}{324.5}=0.013$$

$$\eta=\frac{hs\left（ T_{max}-T_{amb} \right）-Q_{0}}{I(1-{10}^{-A})}=\frac{0.013\times30.4-0.030}{2\times(1-{10}^{-0.313})}\times100\%=35.4\%$$

**S1.5. Serum stability test**

The SPIO@PAsp-DAFe/PEG nanocomposites (4.0 mM Fe concentration), a 20% (v/v) fetal bovine serum (FBS), and a phosphate-buffered saline (PBS) were mixed at 37 ℃ for 1 h, 4 h, and 24 h. 200 μL samples were added to 1 mL ultrapure water in the colorimetric dishes before running DLS.

**S1.6. Intracellular uptake assay**

The amount of Fe^3+^ in 4T1 cells was evaluated. Cells were cultured in 6-well plates (2 × 10^6^ per well) for 24 h and then incubated with SPIO@PAsp-DAFe/PEG nanocomposites (4.0 mM Fe concentration) for another 6 h. After that, the cells were washed with PBS, trypsinized, lyophilized, weighted, and digested with HNO_3_ (200 μL). Finally, the intracellular Fe^3+^ concentration was measured by ICP-MS.

**S2. Supplementary Figure and Table section**


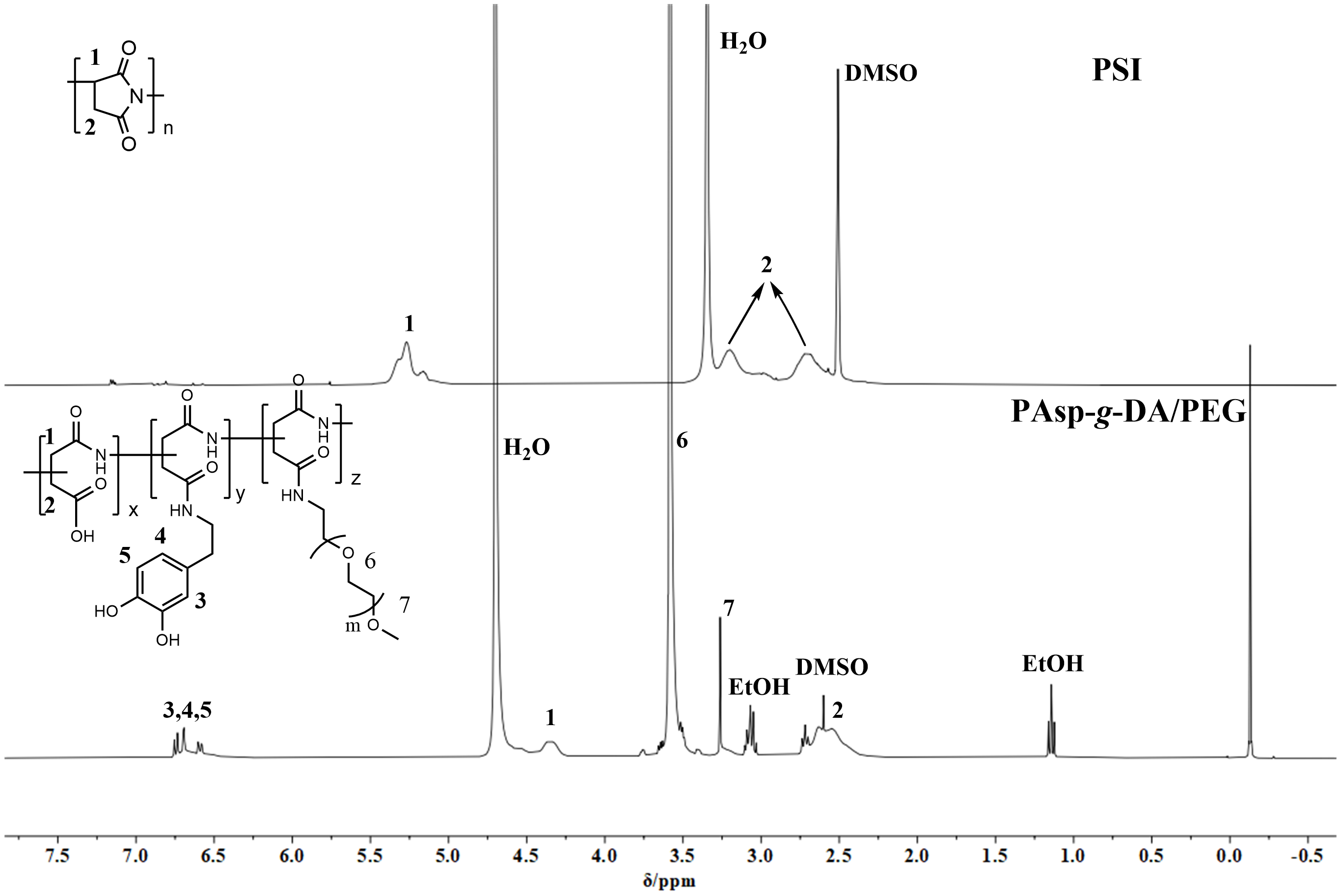


**Figure S1.** ^1^H NMR spectra and characteristic peak labeling of PSI (DMSO) and PAsp-*g*-DA/PEG (D_2_O).


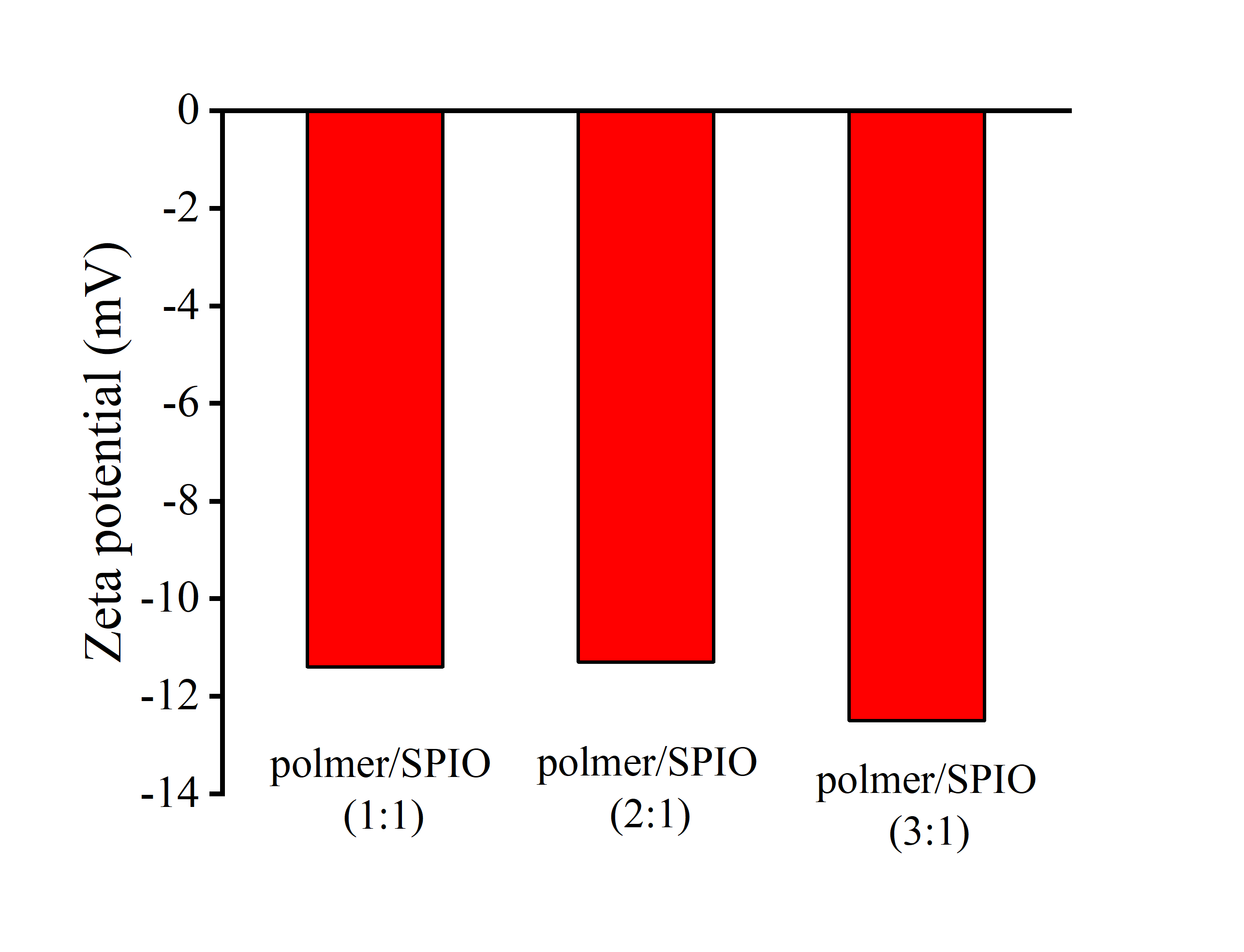


**Figure S2.** Zeta-potential of SPIO@PAsp-DAFe/PEG nanocomposites at different proportions (polymer/SPIO = 1:1, 2:1, 3:1).


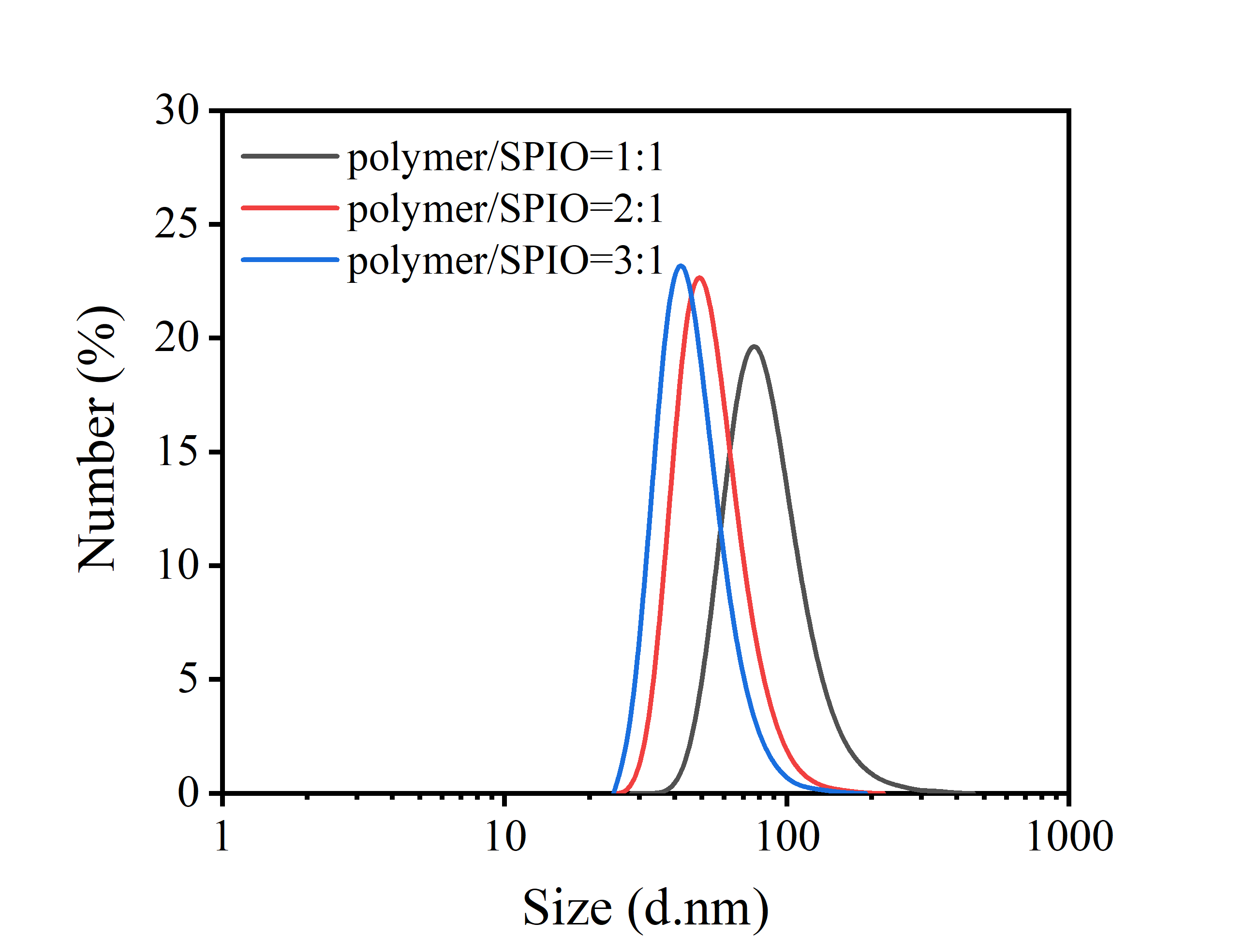


**Figure S3.** Hydrodynamic sizes of SPIO@PAsp-DAFe/PEG nanocomposites synthesized at different proportions (polymer/SPIO = 1:1, 2:1, 3:1).


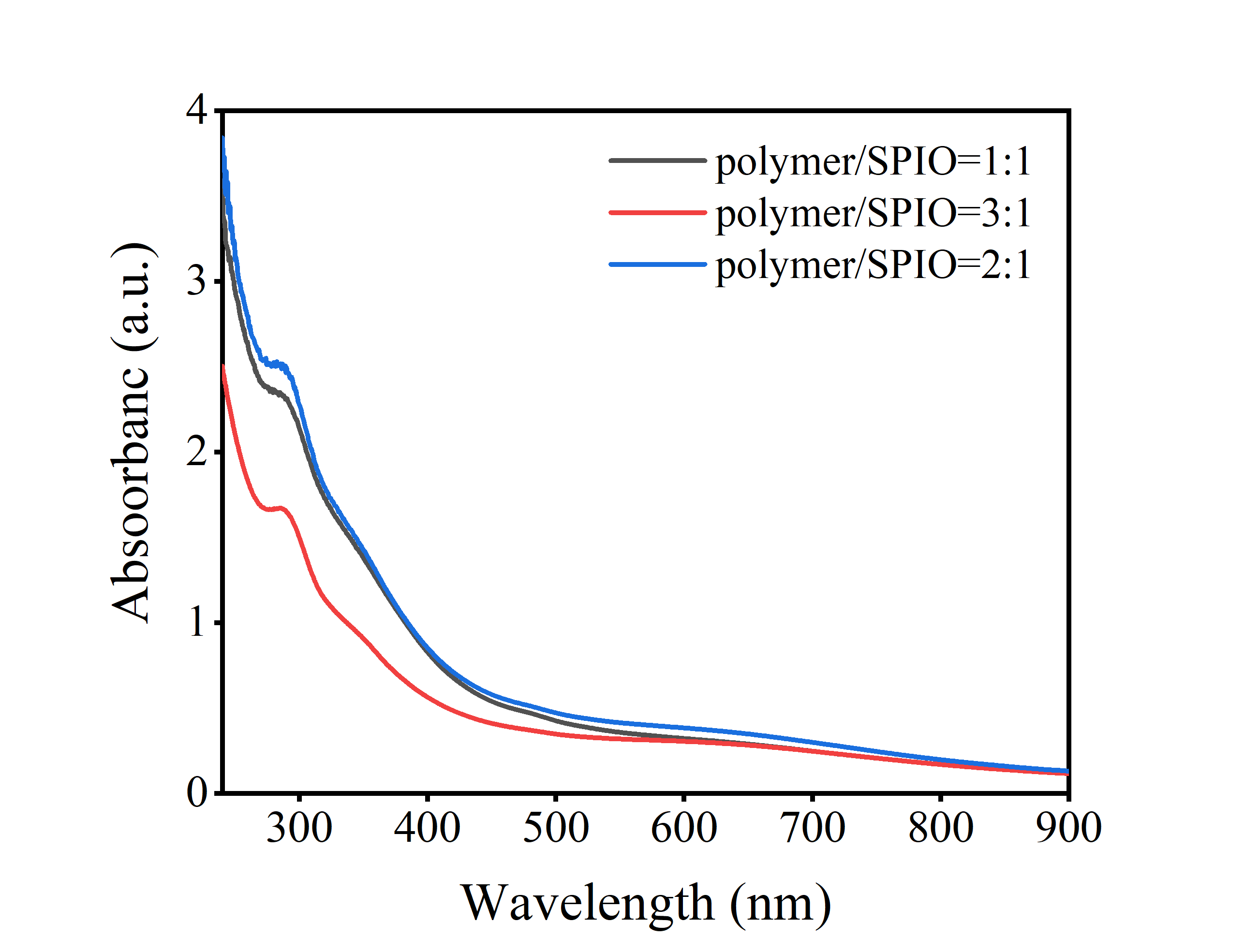


**Figure S4.** UV-Vis-NIR spectra of SPIO@PAsp-DAFe/PEG nanocomposites synthesized at different ratios (polymer/SPIO = 1:1, 2:1, 3:1).


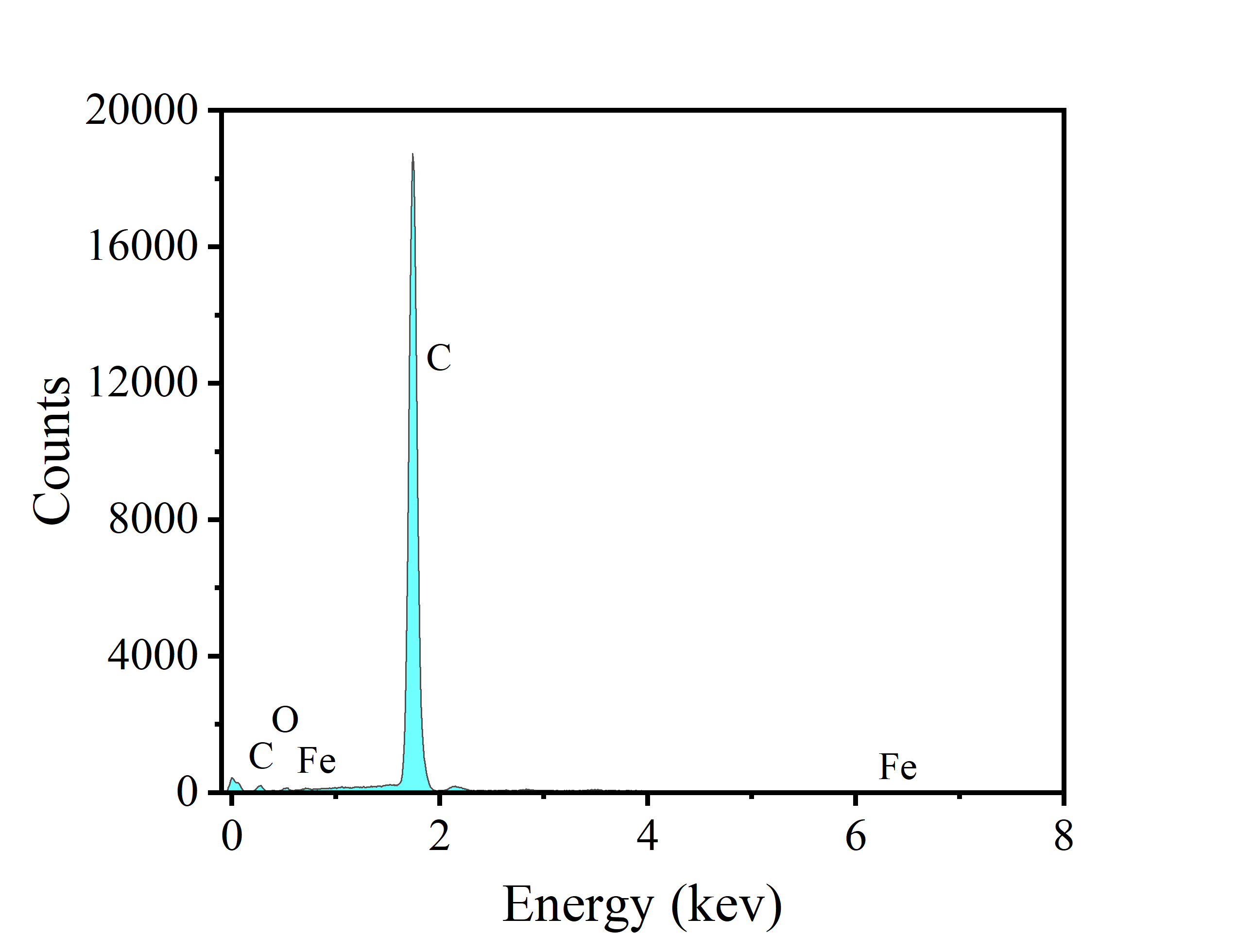


**Figure S5.** Energy-dispersive X-ray spectroscopy of SPIO@PAsp-DAFe/PEG nanocomposites.


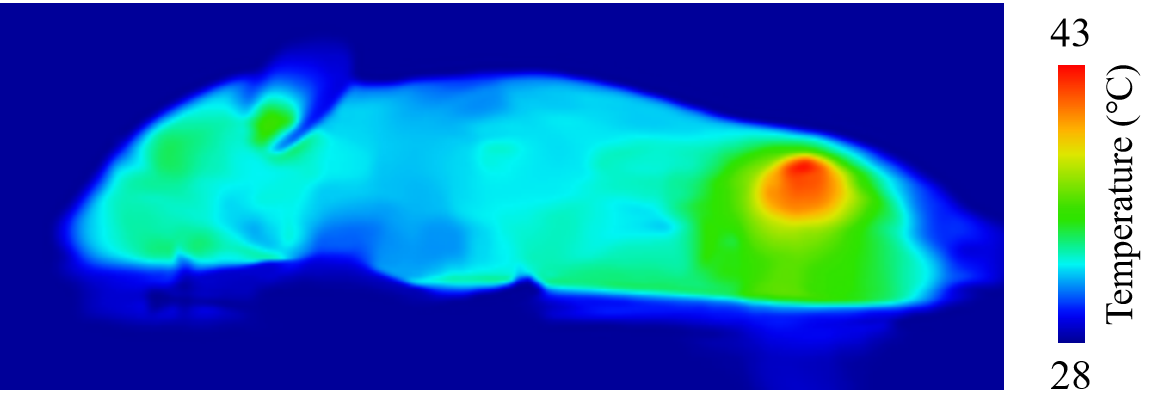


**Figure S6.** Infrared image of the mice tumor heated to 42 ℃ by an 808 nm laser.


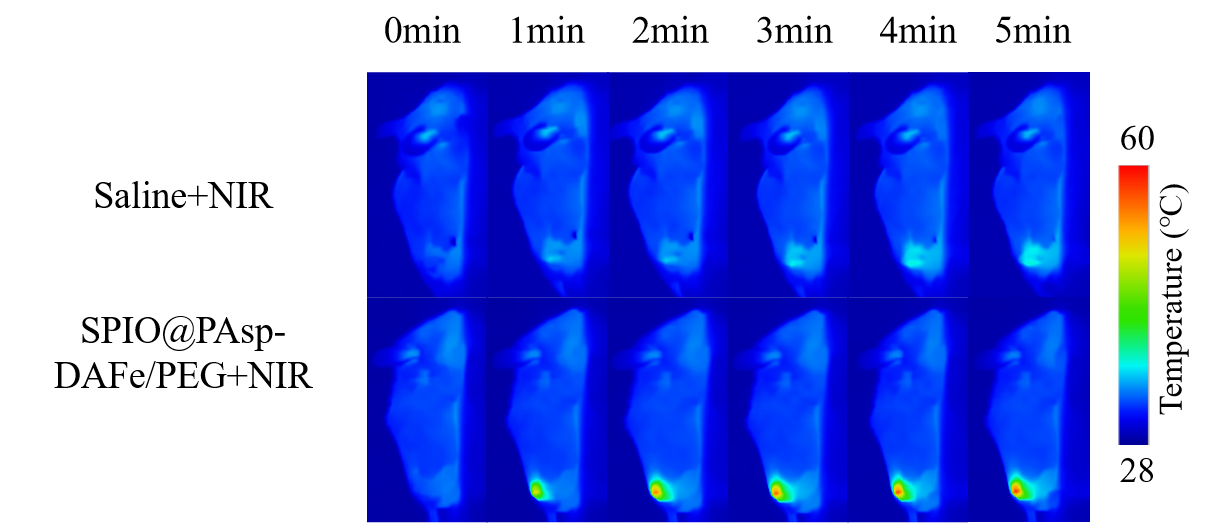


**Figure S7.** Infrared thermal images of 4T1 tumor-bearing Balb/c mice injected with saline and SPIO@PAsp-DAFe/PEG nanocomposites under an 808 nm laser (1.0 W/cm^2^) for 5 min.


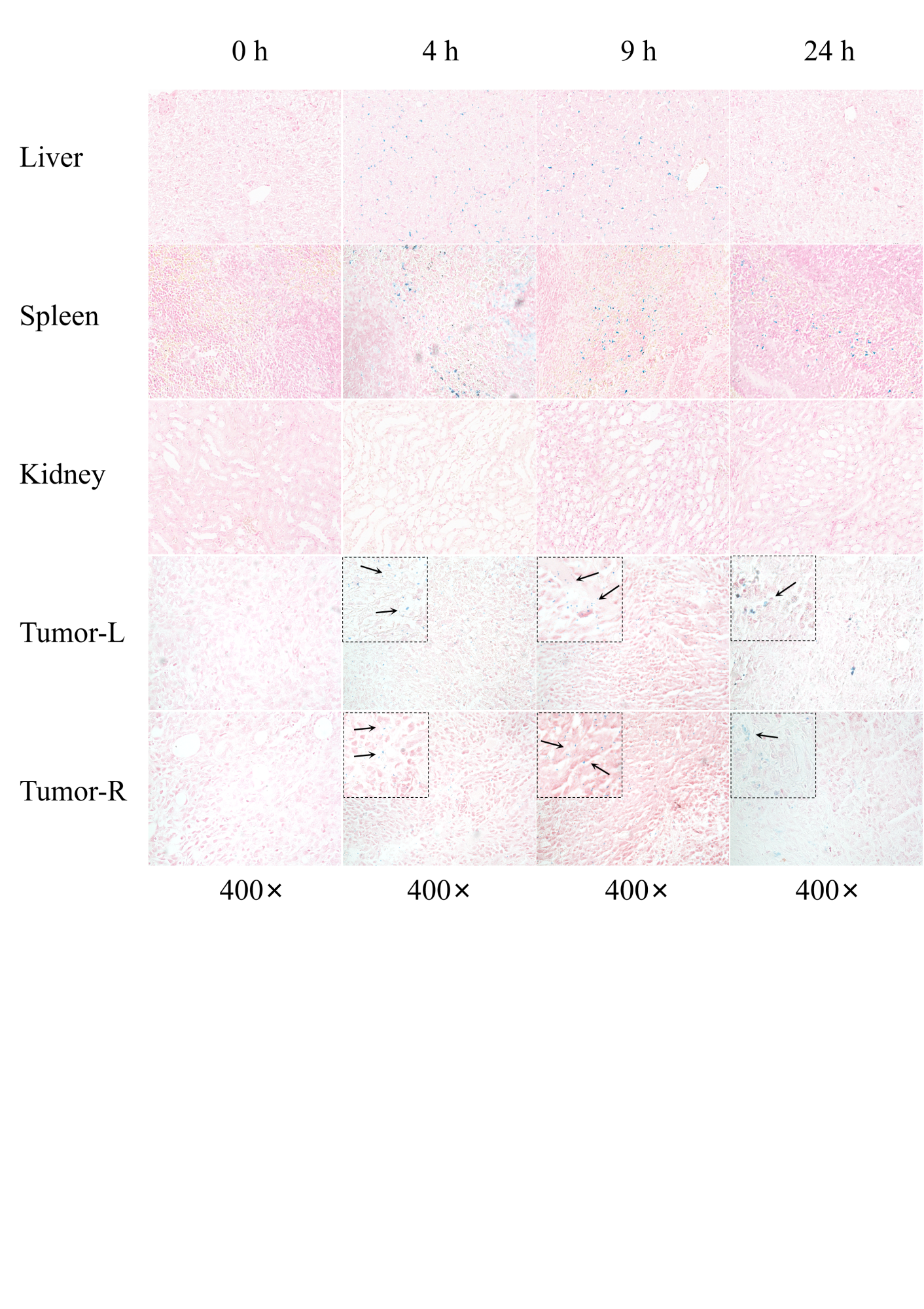


**Figure S8.** Prussian blue staining histological slides of tumor from the 4T1 tumor-bearing Balb/c mice after various treatments and sacrificed on day 15.


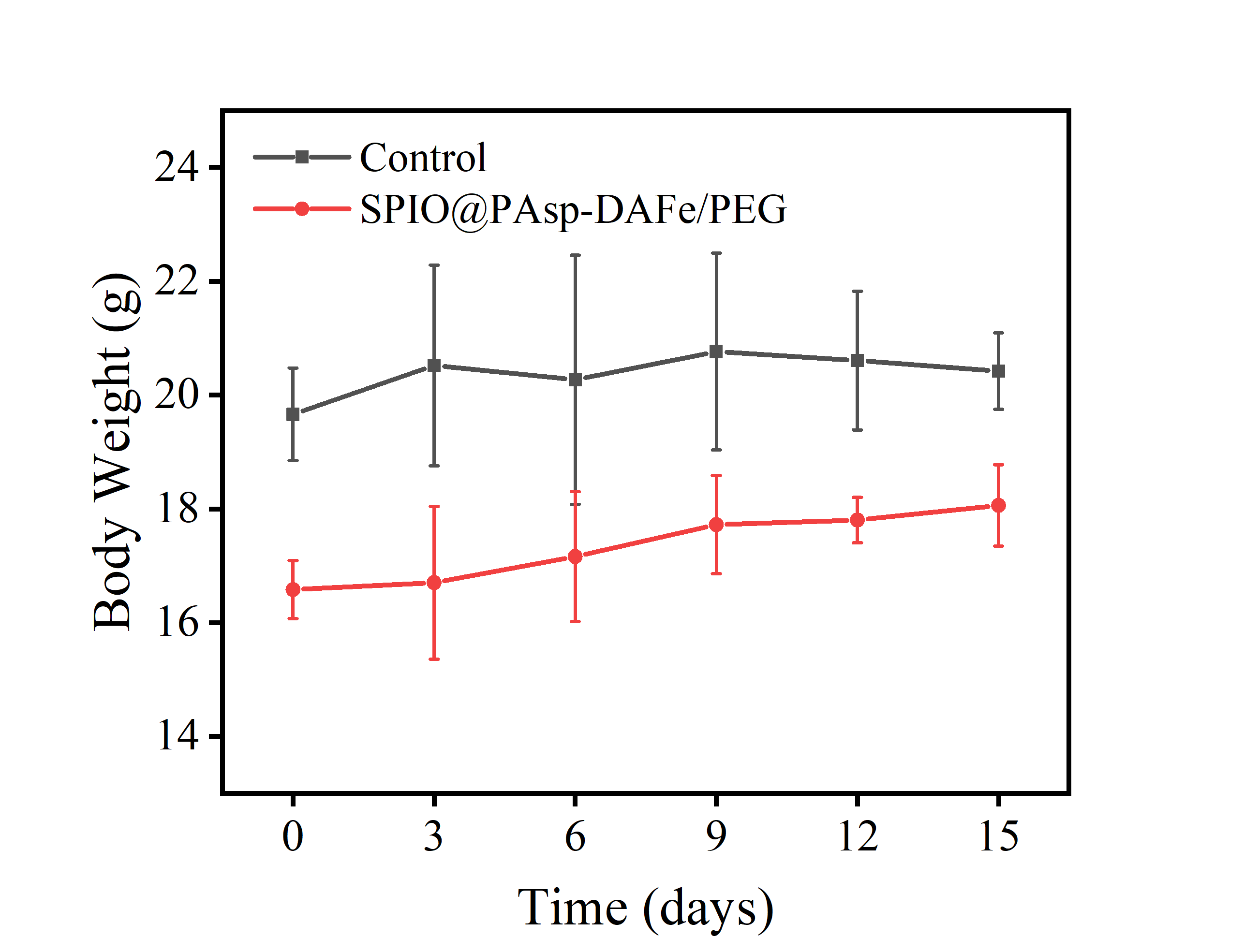


**Figure S9.** Body weight changes of the mice with and without intravenous injection of SPIO@PAsp-DAFe/PEG nanocomposites (20 mg/kg).

**Table S1.** *T*_1_ and *T*_2_ relaxivity of the sample.


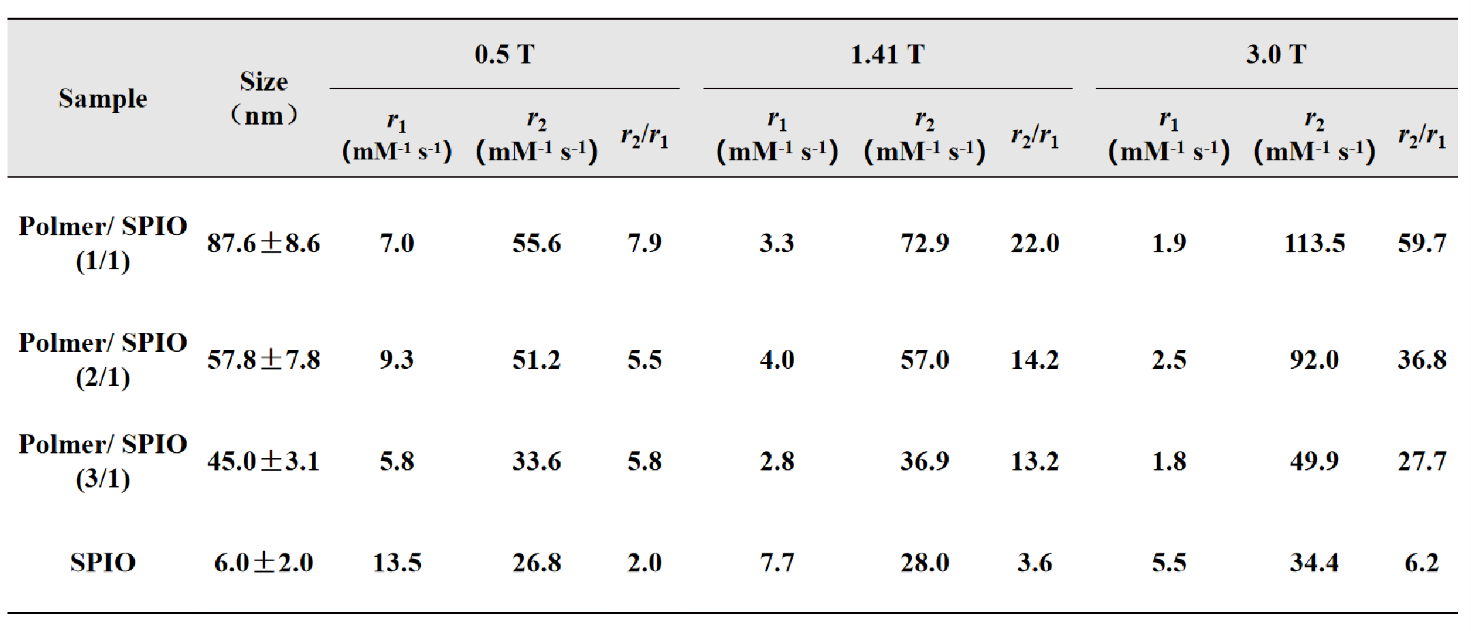

Supplement: rbad022_Supplementary_Data [file rbad022_supplementary_data.docx]
